# Perceptions of the COVID-19 pandemic: a qualitative study with South African adults

**DOI:** 10.1186/s12889-023-15450-z

**Published:** 2023-04-12

**Authors:** Takana M Silubonde, Lucia Knight, Shane A Norris, Alastair van Heerden, Susan Goldstein, Catherine E Draper

**Affiliations:** 1 SAMRC/Wits Developmental Pathways for Health Research Unit, Faculty of Health Sciences, University of the Witwatersrand, Johannesburg, 2000 South Africa; 2 School of Human Development and Health, University of Southampton, Southampton, S016 6YD UK; 3 Division of Social and Behavioural Sciences, School of Public Health and Family Medicine, University of Cape Town, Cape Town, South Africa; 4 School of Public Health, University of the Western Cape, Bellville, South Africa; 5 Center for Community Based Research, Human and Social Development Research Programme, Human Sciences Research Council, Sweetwaters, South Africa; 6 SAMRC Centre for Health Economics and Decision Science – PRICELESS SA, School of Public Health, University of Witwatersrand, Johannesburg, South Africa

**Keywords:** COVID-19, Coronavirus, Coronavirus pandemic, Perceptions, Understanding

## Abstract

**Introduction:**

In South Africa, public perceptions of the COVID-19 pandemic and risk mitigation measures remain mixed. To better understand health behaviours in the context of COVID-19, a qualitative study was conducted, which aimed to investigate perceptions relating to the COVID-19 pandemic among the South African adult population.

**Methods:**

Twelve online focus groups were conducted across the following age groups: 18–34, 35–54, 55 + years old (total n = 70) in December 2021. Diversity across socioeconomic status, geographical areas, and urban and rural settings was maximised, with an equal representation of men and women. Focus groups were conducted, and audio recorded using an online platform, transcribed verbatim and thematically analysed using MAXQDA.

**Results:**

There were mixed perceptions around the pandemic, however, the majority of participants appreciated government actions at the onset of the pandemic and as a result government trust was reported to have initially been high. Nevertheless, as the pandemic progressed, challenges relating to government communication around the pandemic, the inconsistent application of preventative measures by government, the use of soldiers to enforce preventative measures, the banning of alcohol and cigarettes, government corruption and the pervasiveness of social media were reported to have eroded government trust, negatively impacting the uptake of preventative measures. Economic and psychological impacts were experienced differently across income groups. Low-income earners, who already had pre-existing economic challenges reported increased psychological and financial strain. While the once cushioned middle class reported an increase in job insecurity accompanied by psychological challenges. High income earners did not report economic challenges but reported being affected psychologically. Though, low-income earners reported an appreciation of the government financial relief afforded to them middle income earners appeared to not have received adequate financial support.

**Conclusion:**

With the existing mistrust of government, there is need for government to leverage existing trusted sources in communities to aid in the implementation of preventative measures. These findings support the development of context specific solutions to address challenges faced at different socioeconomic levels.

**Supplementary Information:**

The online version contains supplementary material available at 10.1186/s12889-023-15450-z.

## Introduction

In South Africa and globally, the COVID-19 pandemic continues to be a challenge for governments and public health agencies around the world [1]. Initially, before the availability of a vaccine, the South African government employed the use of behavioural, environmental, social and systems interventions such as mask wearing and social distancing measures to curb the spread of the virus [2]. However, from 17 to 2021 vaccines became available to health care workers in the form of a trial and then to the general public from 17 to 2021 [3]. The roll out was staged with access and priority being given to the oldest age groups, with 18–34-year-olds having access to the vaccine in August 2021 [3].

Public perceptions of the pandemic and risk mitigation measures remain mixed, including some members of society still denying the existence of the virus [4]. Moreover, though 86% of respondents in the Partnership for Evidence-Based Response to COVID-19 (PERC) study in February 2021 reported they were aware of the national risk of COVID-19, approximately half of the respondents perceived that they were not personally at risk of the COVID-19 pandemic [5]. These mixed risk perceptions create several challenges for public health prevention strategies, and are exacerbated by the prevalence of misinformation spread on the internet and social media [6]. Conspiracy theories and other rumours about the origin and existence of COVID-19 have spread from the onset of the pandemic.[7–9] This worldwide spread of misinformation has been coined an ‘infodemic’ by the United Nations and has been linked to vaccine hesitancy [7]. Vaccine hesitancy is not a new phenomenon in South Africa. For instance, in the 2009 national and provincial Expanded Programme on Immunization, vaccine hesitancy was reported to be a significant challenge [8]. Moreover, a South Africa measles outbreak in 2003 and 2011 was also associated with vaccine hesitancy [9, 10].

It has also been documented that the precautionary measures implemented in response to COVID-19 have had significant social, economic and psychological impacts [11, 12]. Furthermore, prior research in South Africa has shown how individuals from lower socioeconomic groups are more prone to poor health [13–15] and may experience difficulty in practising COVID-19 preventative measures such as social distancing [14, 19]. An understanding of experiences of the public with these measures are vital in understanding their compliance, so that these can be considered in preparation for future pandemics or other global crises, such as the impacts of climate change. As such, there have been numerous studies conducted in South Africa about the COVID-19 pandemic. The National Income Dynamics Survey – Coronavirus Rapid Mobile Survey (NIDS-CRAM, https://cramsurvey.org) highlighted the social, economic and mental health impacts of the pandemic [16]. A South African study also revealed that people’s trust and satisfaction with political leadership and institutions can be strongly associated with their views on vaccination [17]. Reflecting this, comments and concerns during the health worker trial, about vaccines being tested on South Africans have been widely shared via social media [18]. Other survey studies have reported on sources for COVID-19 information, communication about COVID-19; and factors associated with vaccine hesitancy [19, 20, 21].

While these surveys represent a nimble response to the need for up-to-date information on perceptions, and the types of quantitative data they provide are valuable for providing a ‘snapshot’ from a large number of diverse participants; given that the uptake of preventative measures remains low, further research is necessary to explore these issues in greater depth. Moreover, few studies have sought to find community endorsed solutions to the challenges faced in implementing preventative measures, yet research has showed a greater uptake of preventative measures when the public is consulted [22]. As such, this in-depth information and involvement of the public in seeking solutions to national health threats, may provide a greater understanding of behaviour as it relates not only to COVID-19 (including vaccines), but it can also provide insight into preparation for dealing with predicted future pandemics [23] and other global crises. There may also be insights that are applicable to other aspects of health behaviour, beyond COVID-19. Moreover, while previous South African studies have provided important data from lower income settings, where the impacts of the pandemic were most dire, the middle-income group remains a relatively unexplored demographic, yet it is important to investigate, in order to comprehensively understand the range of responses to a pandemic in an inequitable country like South Africa. The aim of this study was to qualitatively investigate perceptions relating to the COVID-19 pandemic, in order to better understand health behaviours in the context of COVID-19. Specifically, this study intended to explore perceptions of: (i) The impact of the COVID-19 pandemic on life in South Africa; (ii) The South African government’s handling of the pandemic and vaccination roll-out; (iii) Information on and communication about COVID-19 prevention measures and the vaccine in the media; and (iv) What could be done to prepare for future pandemics?

## Methods

### Study design

This qualitative exploratory study used focus group methodology to achieve in-depth investigation. Focus groups were chosen instead of individual in-depth interviews in order to reach a broader sample across a diverse range of settings, and also to provide an opportunity for potential group trends and social norms (according to age group, socioeconomic status, and setting) to emerge in the discussions [24]. To access a diverse sample of participants for the online focus groups we partnered with MoyaResearch (https://moya.app/research-panel/). An online platform was preferred as a tool for data collection because this study took place during South Africa’s fourth wave of COVID-19 (December 2021), a period which was dominated by the highly contagious Omicron variant; this made it impossible to coordinate in-person groups in a safe manner. The focus groups were conducted using the Datafree (https://datafr.ee) video conference platform, Veedo (https://veedo.live), which is reverse billed for participants (i.e. participants do not incur data costs), and allows video and audio recording. Given the high costs of data in South Africa, this platform removed data access as a barrier to participation.

### Participants and recruitment

Participants were recruited according to inclusion criteria drawing upon a national database of potential participants (~ 135,000 individuals, recruited from the larger Moya audience of 6.5 million monthly active users). Twelve focus groups, with six participants per group, were planned, with four focus groups in each of the following age groups: 18–34, 35–54, 55 + years old. We aimed to maximise diversity between the groups in terms of socioeconomic status, geographical areas, and urban and rural settings (and hence home language and ethnicity), although recruitment was more successful in certain provinces. Socioeconomic status was determined by the Living Standards Measure (LSM), which is a segmentation tool that is widely used in South Africa. It comprises 10 groups, with 10 being the highest living standard level, and one the lowest. LSM 5–10 were included in this study. Given the online nature of the focus groups, it was not feasible to include LSM 1–4 (lowest living standard groups) given the challenges of network connectivity and access to a smart phone or computer that would be required to participate in an online focus group.

Information about the focus groups was sent out electronically by MoyaResearch to all potential participants in their national database. Individuals who responded positively were then vetted according to these criteria and contacted to confirm their availability for a focus group. A total of 70 participants ultimately participated in the focus groups, with 53% of participants identifying as female, and 47% identifying as male. After the focus groups participants were reimbursed approximately $5.50 for their time. Reimbursement was sent through mobile money services. Details of the focus groups are outlined in Table 1.

**Table 1:** Details of the focus groups

Group	Age group (years)	Living Standards Measure	Setting	Province	N
1	18–34	5–7	Urban	Gauteng	6
2	18–34	5–7	Rural	Limpopo	6
3	18–34	8–10	Urban	Gauteng	8
4	18–34	8–10	Urban	Western Cape	5
5	35–54	5–7	Urban	Western Cape	4
6	35–54	5–7	Rural	Kwazulu-Natal	4
7	35–54	8–10	Urban	Gauteng	8
8	35–54	8–10	Urban	Kwazulu-Natal	6
9	55+	5–7	Rural	Eastern Cape	3
10	55+	5–7	Urban	Gauteng	6
11	55+	8–10	Urban	Gauteng	7
12	55+	8–10	Urban	Western Cape	7

### Data collection

Twelve online focus groups were conducted, ranging in length from 50 to 103 min in length. Online focus groups were conducted by a research assistant familiar with conducting online focus groups and with knowledge of local languages. All focus groups were supervised and monitored by T.S, the first author. The questions covered participants’ knowledge about COVID-19 and their understanding of COVID-19 vaccines, as well as their perceptions of the pandemic, changes in their lifestyle and behaviours due to the pandemic, the South African government’s handling of the pandemic (including vaccine roll-out), communication about COVID-19 and the vaccines (including media) and preparing for future pandemics. The discussions also covered participants perceptions, attitudes, beliefs, and feelings about the vaccines. Most focus group discussions were facilitated in English; two were facilitated in vernacular languages (isiZulu and isiXhosa) for groups conducted with participants from rural settings. The focus groups were audio and video recorded, translated and transcribed verbatim by experienced research assistants. All participants gave verbal and written informed consent to participate in the focus groups. Ethical approval for the study was obtained from the Human Research Ethics Committee (Non-Medical) at the University of the Witwatersrand (Ref: H21/10/06).

### Data management and analysis

The first author led the data analysis. Transcripts were analysed using thematic analysis [25]. The analysis started with familiarisation with the data through multiple readings of the transcripts. The first author then coded the transcripts and proceeded to organise and sort the codes into themes, in consultation with the co-authors. Data analysis was an iterative process, which involved continuously reviewing and refining themes. This permitted for identification of ideas, making observations, and getting insights and inferences. Coding was conducted until no further codes were identified. Indexing and charting of the data involved merging of the codes into patterns of similarities and differences and aligning them to particular themes and sub-themes. After the coding process, a list of themes was compiled by the first author and key themes (with sub-themes); these were refined in discussion with the other co-authors, and then the codes were applied to the manuscripts with the assistance of MAXQDA 2022 software (VERBI Software 2021).

## Results

The main themes identified were: (1) general perceptions of the pandemic and preventative measures; (2) economic and employment impacts; (3) educational impacts of the pandemic; (4) family and social issues; (5) emotional, social, and psychological impacts of the pandemic; (6) media during the pandemic; (7) government handling of the pandemic.; (8) healthcare and other government systems; and (9) vaccination (10) recommendations for COVID-19 and future pandemics.

### 1. General perceptions of the pandemic and preventative measures

There were mixed views regarding the authenticity of the pandemic. Mainly participants who had not had personal experience with the virus reported scepticism, and either believed that the COVID-19 pandemic had been created by man or that it was simply made up. Others, because they had not seen individuals in their own communities affected, thought the virus only affected ‘white’ people or high-income groups.

*‘It’s a white person’s disease, not a black person’s disease. I don’t know where they got that from because black people will also die. But they say in their areas, people are not going for vaccination, because it’s a white person’s disease.’ Group 7 (LSM 8–10)*.

Another person noted: *‘…in my community, they feel like it was a ruse,’ Group 10 (LSM 5–7)*.

Additionally, participants who expressed low trust in authority and questioned scientific information felt that the pandemic was being over amplified and being used as a political tool to control citizens. However, those who had lost loved ones or had been infected by the virus reported how their scepticism had changed to belief after their personal encounters. These mixed views around the COVID-19 pandemic seemed to affect the uptake of the government measures put in place. Some felt that the measures were cumbersome, unnecessary, and infringed on the rights of citizens.

*‘By taking away people’s freedom of movement, uh it’s like them saying that we have control over you, and the things that you want to do will be at a cost.’ Group 1 (LSM 5–7)*.

Many lower-income residents reported that their communities had not been complying with the measures, because of their negative economic impact. In contrast, in the high-income communities, participants reported how they had united and supported one another, ensuring compliance of the measures. The high-income groups also relayed how they felt the measures should become a way of life and not be removed.

*‘On the bright side, if you’re looking generally, we are a more hygienic nation now, wash our hands more often, we sanitize if you go to the shops, at least now you getting you know you to sanitize the trolleys. I think there’s more hygiene around. We have adjusted in the new way of doing things.’ Group 12 (LSM 8–10)*.

### 2. Economic and employment impacts of the pandemic

As highlighted above, the income level of participants seemed to influence adherence to measures. The negative economic impact of the pandemic was hugely felt among the low-income earners, where unemployment was high but increased dramatically with COVID-19. Hence, the government subsidy given was hugely appreciated by this income group.

*‘Yes, first of all we received R350, our kids don’t work, and they received R350 and then at least things were a little better because there is no work.’ Group 6 (LSM 5–6)*.

The middle-income group reported the detrimental effects that the pandemic had on employment. Many, especially those in service jobs, reported retrenchments or having received a pay cut. This resulted in some selling their homes under duress, lowering their quality of life, and having to remove their children from their schools of choice.

*‘Okay on my side it affected me a lot because I was working and when the pandemic started, I was retrenched. I am currently the bread winner at home so, things changed from where I was to where I am right now. It just made things very difficult because then I knew every month something was coming in and then the pandemic started, things had to stop. Because I have kids, the school fees I couldn’t afford.’ Group 3 (LSM 8–10)*.

On the contrary, some participants, mainly in the high-income group, mentioned that the pandemic had not affected them much. Many in the technology sector or in management stated that nothing much had changed for them financially during the pandemic. A psychologist reported that because of the pandemic, his business had grown exponentially as there was a demand for his services.*‘*.

*‘Okay, personally, uhm, not much has changed for me, other than my practice has exploded exponentially,’ Group 11 (LSM 8–10)*.

Working from home was a challenge for many participants. Difficulties achieving a work-life balance *“when your place of rest had also become your office*” were reported. With the move to virtual meetings, participants reported the negative effect that back-to-back meetings had had on them. For parents, balancing work and home-schooling was reported to have been stressful.

*‘When the pandemic started, we had to work from home, schools shut down and we had to do home-schooling, which was a lot for us. As a family we have got two children. My husband was teaching my son, I was teaching my daughter, I had meetings all day, obviously now because we’re working from home’ Group 2 (LSM 5–7)*.

### 3 & 4 Education impacts of the pandemic and family issues

Participant responses indicated large inequities in the impact on education. All children missed school, but some were able to do online or home schooling while many in the low-income group did not have access to the facilities or time for home-schooling. *‘For instance, my nephew didn’t miss a day of school but his friends in townships have lost a whole year.’ Group 8 (LSM 8–10)*.

They were fears that children in this income group would not return to school or had been further left behind by their high-income peers, affecting their educational and economic futures. Those in higher education reported delays in graduation, internships, and difficulties in studying from home.

*‘I was supposed to graduate like last year but because of Corona I didn’t graduate, so it affected me so badly, so now I can’t get employed, I need HR internship, so I can’t get it because of Corona…’ Group 1 (LSM 5–7)*.

On the one hand, a few participants said that lockdown had created a good environment to be together with family members and had enabled productive use of time. On the other hand, some higher income participants described their isolation and loneliness that resulted from the pandemic. They complained about how the lockdowns and social distancing measures had affected their lifestyles, as they were unable to travel, attend weddings, go out to malls and parties.

*‘We used to just enjoy taking long weekends away with the family and the kids. But I think we’ve toned that down, we just became sceptical of travelling, we prefer being at home and just going close but not sleeping away. So, we are at home most of the time.’ Group 8 (LSM 8–10)*.

For many middle-income earners, the lockdowns had resulted in those working in different provinces where their immediate families lived, causing them to being separated for months longer than usual.

*‘I was used to go home every two weeks so now due to this COVID I can’t go home at all, so hey I’m struggling, yes, you must imagine spending the whole 2 months without seeing your son.’ Group 6 (LSM 5–7)*.

Those with family living abroad could not travel to see each other, and in some instances some participants had not been able to bury loved ones. Additionally, some reported not interacting with elderly family members, as they feared to infect them. Although there seemed to be an appreciation of the need for these measures, these participants’ responses indicated how isolation and loneliness increased as a result of these circumstances.

*‘I have got 3 kids, one lives here in Sandton, I live in Fourways, but in the last 20 months, we have only seen him about 3 times, because he doesn’t want to interact with us, we are protected very much, one kid is in London, and we haven’t seen her for the last 2 years.’ Group 9 (LSM 5–7)*.

### 5. Emotional, social, and psychological impacts of the pandemic

Due to the psychological stress caused by the negative economic impact of the pandemic, and family and social issues that participants faced, the pandemic was reported to be emotionally taxing. The anxiety reported by the low-income groups was related to the additional financial strain caused by the pandemic. While the high income reported loneliness from isolation. When asked about the psychological impact of the pandemic one participant gave the following response:

*‘I would say the big, big lockdown because we had one afterwards which was a little smaller but obviously there was a lot of heaviness around. Everybody was feeling a little heavy and unsure of everything, we kind of learnt to get through it. I think for a lot of people, even though everything lifted, and lot of the restrictions lifted, for a lot of people that heaviness stayed with them.’ Group 8 (LSM 8–10)*.

Many middle-income earners reported struggling with fear and anxiety as life had become uncertain. As a result of the pandemic, participants reported that they were unable to plan for the future, and that when they did plan, they were often met with disappointment. Fears around interacting with others, and future employment opportunities were reported. For some, this fear had turned into paranoia.

*‘I think life has become very scary lately, we are even afraid to go and visit our aunts, our sisters because every time you cough you are worried, what if I have Covid; so life became scary by the minute and we are so scared, we don’t even know what our future holds at this moment, we are not even sure if we’re going to get jobs after this, if uh our economy will survive this, we’re so afraid at this moment, personally I am afraid.’ Group 1(LSM 5–7)*.

### 6. Media during the pandemic: ***Social media and other media***

The reported most common source of COVID-19 information in their communities was social media (WhatsApp, TikTok, YouTube, Instagram, and Facebook). Social media information was said to be easily accessible and easy to spread, as it often just came to you without effort (e.g., forwarded WhatsApp messages).

*‘…they are passing on information without verifying or certifying that the source of information is factual and true, and I think people have mentioned about Facebook and WhatsApp or whatever the case is. So, it’s very quick to pass a message on, but it’s fake news. It’s not true.’ Group 10 (LSM 5–7)*.

Participants highlighted the fear that was propagated via social media and how they spread social media information without verifying its authenticity. Though both young and old were influenced by social media, the young were seen to be more susceptible to it.

*‘So, with the information that has been spreading through social media, it has been quite challenging for our people to understand this. The Corona virus eventually looked big, whereby you’d see people saying there’s this Corona virus it has been planted to eliminate the population.’ Group 5 (LSM 5–7)*.

*‘They do not research, they don’t go further and try to understand that is it really like that, or we just take whatever we find. If I can just find the first information I get, I just use that. That is why you have too many stories that are trending. I think social media is the most important one that these days because you’ll find that everything happens on TikTok. People are listening to the media, to social media, and then reacting to that. If they say something will kill you, people believe it.’ Group 4 (LSM 8–10)*.

*‘Most young people rely on social media more than anything else and from what I’ve seen, since the beginning of the pandemic, a lot of them are being ignorant.’ Group 5(LSM 5–7)*.

Both young and old participants stated that they did not trust social media, but at the same time highlighted the accuracy that social media had in predicting government decisions. Some mentioned how social media was largely influenced by the West and was responsible for polarising people.

*‘ it’s these videos that come from overseas, for instance other videos they will come from England they will come from the United States because all these big power countries they still have that belief that they can still control the world; so their social media is more stronger than our social media because uh they are big countries; so those scientists, they are scientists who are saying one thing and our scientists are saying something else…’ Group 11 (LSM 8–10)*.

Participants highlighted how social media was often believable as there was usually a trusted ‘face’ behind it, unlike government information. Social media exacerbated the uncertainty and fear surrounding the pandemic. Some participants reported that in order to make informed decisions they did their own research and avoided social media.

*‘It’s so difficult to distinguish (between true and false) because other people show their faces and present something which is not true? For instance, I’m talking about the vaccination there are people who say vaccination is not good, you will die in two years’ time. And those people show their faces. And on the other side, government says vaccination is the good thing. So, you end up being skeptical? You don’t know you don’t. You are uncertain whether this is true.’ Group 2 (LSM 5–7)*.

*‘Look social media didn’t affect me as much, I am more of a factual person, so I went on the net mostly. The W.H.O (World Health Organisation) I only found out about…… Which then gave me more insight. But then part of me then gave me that – you know what I am a bit of a spiritual religious person as well, part of me was like this might be the second coming, this is the mark of the beast, you know. But then I needed to be scientific, come down to earth in this case.’ Group 8 (LSM 8–10)*.

Most participants had a positive perception of television, print and radio information. With participants expressing a greater trust in international news sources.

*‘I usually get that (reliable information) through radio, print, like newspapers…. Not social media information, because that is filled with many, many manipulations. So, I did not listen, or put it into mind the information I get on social media.’ Group 6 (LSM 5–7)*.

*‘I would say the more international news I would trust more because I feel that they are not so much influenced politically…’ Group 9 (LSM 5–7)*.

### 7. Government handling of the pandemic

#### Government communication

The majority of participants appreciated the ‘family meetings’ initially held by the South African President, where, in a national TV presentation he would clearly state the plans of the government to mitigate the spread of the virus. The “meetings” had fostered unity among citizens and had resulted in the government being trusted.

*‘I think what the government did quite well was communication. Communication was there, as we are all aware that the President was constantly calling on meetings and updating the nation on what was happening. I think that was handled quite well, at the beginning.’ Group 5(LSM 5–7)*.

*‘That is a big one, I would say in the beginning we were spot on, out of all the countries. Put it this way out of all the countries I was grateful I was in South Africa. When the first lockdown came, I thought the xxx (president) did an amazing job. Group 8 (LSM 8–10)*.

However, some viewed government predictions and actions at the onset of the pandemic to have been inaccurate and driven by fear rather than facts.

*‘You will recall that COVID was started on like, serious misinformation. For example, at the very beginning, it was predicted that between 80,000 to a 100,000 people would perish based on people’s comorbidities…. But today, we’re on the first wave and we are yet to reach 100,000 deaths. What is evident, is our government that is panicking, and really ready to crumble, and just wasting money……They have totally failed. Absolutely failed. They’ve crashed an economy that was already in junk status.’ Group 12 (LSM 8–10)*.

Of great concern was the government’s lack of response to misinformation on social media, and the inadequate government use of social media to disseminate accurate information was highlighted.

*‘And government is not countering the conspiracy theories enough for example about the chips that are being implanted in people.’ Group 9 (LSM 5–7).*

Additionally, the government was reported to have contributed to government mistrust by giving contradictory information which propagated fear in communities.

*‘I think I would say that, government themselves, they need to be on the same page themselves, cause they seem to be contradicting themselves most of the time, and they should stop this thing with scaring people and scaring all of us with this thing like saying in May, there will be a new wave and in September, how do they know, so those kind of things, they make us wonder, really, it this thing really real, or is it really man made as people say.’ Group 6 (LSM 5–7)*.

However, some participants stated that government had communicated effectively, but were not a trusted source of information on COVID-19 matters.

*‘…. there’s been vaccines problems in South Africa partly because the people don’t believe the government.’ Group 9 (LSM 5–7)*.

*‘They are the people that are supposed to inform us, however they themselves, they are not informed or what, I don’t know, but someone somewhere in government needs to make a decision and they need also themselves to be educated, so that we can find out, what is it, really, that is happening around us.’ Group 7 (LSM 8–10)*.

Moreover, the removal of a prominent scientist, as a government spokesperson, had raised some suspicion among participants.

*‘…there was this professor who was leading this initiative, she had to quit because she was in disagreement with some of the findings, the name was xxx, I think. She had to resign because the government had other opinion.’ Group 12 (LSM 8–10)*.

#### Implementation of COVID-19 pandemic measures

Initially, government was perceived to have implemented COVID-19 pandemic measures well. Many participants praised government actions and believed that it was the citizens who were letting the government down. However, the effectiveness of the implementation of measures was reported to have declined with time, with participants calling for stricter implementation.

*‘I think some time ago, there was this thing that said that is if you’re found in public without your mask, you could get arrested or something. I think they should bring that back because right now people are going all over in public not wearing their masks. I feel like that should be bought back.’ Group 4 (LSM 8–10)*.

Governmental trust was reported to have declined as the pandemic progressed and the main reason for the decline was government corruption. It was apparent to the participants that “the government officials did not care about the people but were after their own gain.” Further “adding salt to injury” for the participants was the government failure to hold the “looters” to book.

*‘I was really disappointed with xxx, the prior Health Minister, I thought he was doing such a great job. When the scandals came out, it was so disappointing…Then what was very disappointing was the corruption of PPE, that whole saga was just disappointing. Here is the government trying to do its best for its country. But again, corruption is letting us down again.’ Group 5 (LSM 5–7)*.

*‘…we need to see more people being brought to book for wrongdoing when it comes to the issue of stealing money earmarked for COVID-19. If we can see that I think people are going to see that this pandemic was taken seriously.’ Group 12 (LSM 8–10)*.

The preventative measures put in place were reported to be selectively applied by the government. Ministers were seen to be having parties while citizens were locked down. Additionally, elections “which would benefit the government”, were being held despite the pandemic. Participants also found it suspicious how cases and the measures were increased as soon as government elections were wrapped up. Furthermore, there were reports that in low-income settings, some community members had been arrested for not following COVID-19 pandemic measures but were later released after paying a bribe.

*‘They themselves (government) are not adhering to the regulations because when they are having their own meetings and events, they flock up wherever they are but expect the public to adhere to all of these regulations. When it is their turn to show or be an example to society itself, they do not adhere to these protocols at all. So, it is something that is really disturbing to see because it looks like at times, we are being taken for granted in a way. I understand that it is a pandemic but at times if you lead people, you must also show an example of leadership as well, you can’t just do as you please just because you are a leader of some sort.’ Group 3 (LSM 8–10)*.

The government was said to have developed good apps and initiatives, however there seemed to be a lack of follow through. Additionally, some viewed the government as incompetent and oppressive, linking measures to past occurrences such as apartheid policies.

*‘Okay, the first thing that I want to focus on is what didn’t work, there used to be an app that you can download, it was the COVID Alert SA app, but nobody bothers to update that app, so you never know where there were infections near you, so I think that thing never took off, it was a dead up from the start.’ Group 9 (LSM 5–7)*.

*‘That is a difficult one. They couldn’t do it with the Zondo commission, TRC (related to apartheid), the arms deal. The government is lagging with everything. They are now implementing (apartheid) laws of arresting people for hate speech, misleading people on social media. It is all about arresting the citizens. They are adding more confusion.’ Group 12 (LSM 8–10)*.

*‘I really don’t see why they needed to close the bottle stores…. I don’t think that was fair on lot of people and to put restrictions on (alcohol), I know they were maybe reflecting on apartheid.’ Group 2(LSM 5–7)*.

### 8. Healthcare and other government systems

The government systems were viewed to have been ailing before the pandemic and hence, did not cope during the pandemic. Participants highlighted how the healthcare infrastructure and the systems in place were inadequate. Furthermore, there were views that when the virus emerged the South African healthcare system failed to prepare. The COVID-19 unemployment grant system was said to be flawed. Many did not receive the money due to them and enquiries were difficult to make. Queues at government offices were reported, this when social distancing was a vital measure. Their online platforms were also viewed as inadequate.

*‘I think because the underlying issues that the government had before the pandemic, it exposed the government because of their faults they couldn’t fix in the past but now those problems are beginning to show during this pandemic.’ Group 2 (LSM 5–7)*.

*‘The health system they can definitely improve on the health system, for example, if you go to a clinic now and you get tested, it will take up to 10, 12 days to get the results back, I mean by that time it doesn’t matter anymore.’ Group 9 (LSM 5–7)*.

*‘As someone who works for government in xxx, for example, there were also a lot of system inefficiencies, our systems were overloaded, Hence, I heard someone here talking about the struggles they came across as they were trying to claim from the xxx. …. The system was flooded, and we were exposed in that we are incapacitated and in our technologies are quite behind.’ Group 5 (LSM 5–7)*.

Furthermore, participants who were frontline workers shared the physical exhaustion and anxiety they experienced. Those in the healthcare sector also highlighted how difficult it was seeing patients die and how their mental health had suffered. There seemed to have been no support provided for these workers by their employers or government.

*‘It’s actually my anxiety went straight through the roof… I think it’s the second wave that hit us all at work because unlike everybody that can work from home, we had to be at work every day. And we were working with sick people all the time. And people that you’ve known all your life because I’m at that surgery for 34 years, the patients I’ve known all my life. Suddenly the mom, the dad and the sister pass away in one week. So, it really it took its toll on us.’ Group 10 (LSM 5–7)*.

### 9. Vaccination

#### Perceptions of vaccine roll-out and vaccines

Participants cited that they appreciated how government had made the vaccine free for all. The vaccine rollout and information on measures were said to have been well advertised in urban areas, with individuals stating that the messaging system and adverts had worked extremely well. However, concerns were raised around the accessibility of the vaccine, and several participants highlighted that they believed that the vaccine rollout was initially a bit slow, which had resulted in some vaccines being wasted. Vaccine sites were reported to be far for some, which meant transportation costs. Some complained about the long queues and not being able to take time off to stand in the vaccine queue. It was also reported that when government was approached, they were unwilling to open sites at workplaces.

*‘I think to a certain extent it was very well publicised, I mean you heard on the radio, you know you would see signs everywhere just driving into the city, it was the talk of the town, so we knew exactly when vaccination started, which date so I don’t think that there will be people out there who didn’t know about the vaccine rollout, so it was really well publicized.’ Group 8 (LSM 8–10)*.

*‘I think they tried to get to most of the rural places, but they don’t seem to be doing it very well. I think they should get more mobile clinics and things because I think the majority, the people that aren’t vaccinated are probably in rural places. They should start doing something, seriously.’ Group 10 (LSM 5–7)*.

*‘The queues are still long, if you go to certain pharmacies, the queues are still long, and private hospitals even, the queues are still long, for as long as the queues are seen to be that long, or not efficient, people are going to withdraw.’ Group 9* (LSM 5–7).

*‘For example, in my workplace, we were talking to the Department of Health to make sure that, to try and organize that they have vaccination stations in the workplace, and the department told us that they don’t do that. So, I feel that the accessibility could be an issue for some people.’ Group 5 (LSM 5–7)*.

It was highlighted that the elderly in the rural areas mostly received information from their family members, as access to media was limited. Additionally, though many felt that they knew information about where to get the vaccine or what measures were to be followed, they expressed that government was forcing individuals to vaccinate without providing citizens with enough information on why they should vaccinate. As a result, it seemed to some, that they were being forced into doing something they did not fully understand.

*‘With the vaccination program…. they do not inform people on why they are getting vaccinated. It is more about either you vaccinate, or you do not. I think they could have provided us with more information on why we should be vaccinated so that people are not frightened about what is put in their bodies.’ Group 3(LSM 8–10)*.

Some were wary about how quickly the vaccine had been made and believed they would suffer future side effects. While others cited concerns around dying because of the vaccine. Many participants believed that social media had fuelled anti- vaccination sentiments, resulting in heightened fears around the vaccine. Furthermore, some reported relating the vaccine to end-times of the bible and hence, were fearful of the consequences of vaccination. Many participants did not want to keep getting ‘boosted’ and feared the possible long-term consequences of continual vaccination.

*‘They must just leave us alone with the vaccine because now I heard there’s a Johnson and Johnson booster shot uh, I’m not for that one; I got a Johnson vaccine but I’m not going back for the booster one.’ Group 1 (LSM 5–7)*.

*‘Okay my first one is that they say we must go and get another dose of the vaccine you know, so now I get scared because it’s as if they don’t trust the first dose that they’ve put in us; so I just think that what will this thing do now if we have to go back and get vaccinated again, and it’s like how many doses will we end up having to get, I mean will it get to maybe 10 or something…’ Group 11 (LSM 8–10)*.

There also appeared to be confusion around the communication on different vaccines. Further complicating the issue, the government focus on some groups to vaccinate had unintentionally sent a message to some that they did not need to vaccinate. Additionally, the communication around the incentives for the elderly to vaccinate had been interpreted by some as a bribe, leading to further suspicion.

*‘I feel like it was not handled quite well because the President started with the older people first. I feel like he should have made it available for everyone because at the moment he made the older people important. He made it look like the youth don’t really need the vaccination. Like, it doesn’t make sense for me to get it right now, because at the beginning, it was for the older people who were ill.’ Group 4 (LSM 8–10)*.

*‘Yes, they must explain the difference between Pfizer and Johnson and Johnson, the differences they should explain it and not include money because in black communities when you include money that means it’s a bribe.’ Group 1 (LSM 5–7)*.

The participants reported a lack of clarity in information given at vaccination sites and clinics, promoting vaccine hesitancy. The fact that some healthcare staff were also vaccine hesitant further fueled vaccine fears. Additionally, the clinics were giving mixed messages on who should get vaccination, causing further confusion. The historically trusted healthcare sector did not seem to communicate with one voice, aggravating the already present fears and mistrust of citizens.

*‘…you go to the vaccination centre; you go there, and you ask for information and still they don’t even know themselves the nurses that you take shots from, what’s this honestly; some of them are even scared of getting the shots themselves, so why do we put our lives in them so that we trust them with our lives.’ Group* 1 (LSM 5–7).

*‘Many of the patients weren’t aware that you can get vaccinated while you were pregnant because you will get different information from different clinics. Some clinics will tell you that it’s dangerous to get vaccinated while pregnant, while others will inform you it’s good to get vaccinated while pregnant, it will protect you and the baby and it is safe, while other clinics won’t do the same.’ Group 6 (LSM 5–7)*.

*‘At the vaccination centres they kind of said that when you’re taking uh two medication, chronic medication you’re not supposed to get vaccinated, so I can’t understand…’ Group 2 (LSM 5–7)*.

The fact that one could still contract COVID-19 even after being vaccinated was a deterrent. There was also a perception that those vaccinated were individuals who were being hospitalized with the Omnicron variant. Others were suspicious of the use of Western vaccines instead of the Russian and Chinese vaccine. Some participants also stated that a South African vaccine would be more trusted. Additionally, the use of traditional herbs during the pandemic was highlighted. Some participants also questioned why one had to continue to wear masks after being vaccinated.

*‘.is why do I have to take the vaccine because it’s not going to prevent me from getting COVID anyway, so what’s the point of taking the vaccine.’ Group 7 (LSM 8–10)*.

*‘…when the rollout started, we spoke of only three companies, which were the AstraZeneca, the Johnson and Johnson, and Pfizer, and then forgot that there were other vaccines that were available on the market, the Sputnik from Russia, and so on. But government chose to be selective as to which vaccine they will be pushing on the people of South Africa, and that on its own left many people skeptical as to the intention of the government.’ Group 5 (LSM 5–7)*.

*‘African wormwood is one of those plants that our grandmother used to tell us about. If you have flu or something, you boil it and drink it like tea. You drink it, then you’ll be fine.’ Group 4 (LSM 8–10)*.

#### Vaccine mandates

There was a great debate surrounding vaccine mandates. While some thought the government should enforce vaccine mandates, others expressed that vaccine mandates infringed on one’s rights. With some further expressing that they felt they were being manipulated into vaccination.

*‘…they must say that if you’re not vaccinated you can’t go into certain places, people must produce confirmation of their vaccination and I think that would help and people would start going to these vaccination sites.’ Group 11 (LSM 8–10)*.

*‘…he should actually make it mandatory. Because if everyone if most of us are vaccinated, it will really help to keep the figures low.’ Group 10 (5–7)*.

*‘… by forcing people and taking away their rights at the end of the day… like for me personally I don’t think you should ever take someone’s right of freedom away (by forcing them to vaccinate) at the cost of something else…. but this is like… post or pre-apartheid people where we’re fighting, we’re fighting against oppression in a sense….’ Group 1 (LSM 5–7)*.

*‘I feel I am now forced to vaccinate, and if I do it, then I’m doing it because they say so. It’s not because I understand the reasons behind vaccinating. It is something that I have to do because I need to get a job, or I need to fly to USA or whatever. I think it is now just more confusing than ever.’ Group 4 (LSM 5–7)*.

### 10. Recommendations for COVID-19 and future pandemics

Participants emphasised that society had a role to play in providing solutions to the pandemic. Citizens were encouraged to comply with government regulations and not only do so when policed. Additionally, citizens were encouraged to correct each other, educate each other and be the ambassadors of the vaccine. Finally, citizens were encouraged to become critical thinkers and to evaluate social media information before sharing.

*‘We really have to start doing more research instead of word of mouth from the street which is not verified.’ Group 12 (LSM 8–10)*.

Participants highlighted the need for the government to prioritise educating the citizens, emulating the intensive education the nation received about HIV. The following methods of education were suggested- door to door campaigns, community workshops as well as engagement of community leaders. In their education campaigns, the government was encouraged to have a targeted approach to communication, according to demographics, that would ensure that all citizens obtained and understood information on the pandemic. The use of scientists and trusted health care professionals to disseminate information was emphasised. The government was encouraged to actively counteract misinformation as well as make use of social media sites to disseminate information. Participants highlighted the need to have platforms where credible information could be easily accessed by the public. Furthermore, participants advised government to involve citizens and the private sector in decision making.

*‘They need to invest in more innovative ways and the only way is to speak to young people, get the young people involved, because they know how to reach the masses through other ways that that are innovative. I don’t think they are doing very good job when it comes to that.’ Group 9 (LSM 5–7)*.

*‘the government must just give information overload and hold workshops, so that people know exactly what is going on and what the vaccines are. Because I remember when this thing, HIV AIDS, when it was still like when it came out. It was introduced at schools; everyone was learning about it.’ Group 2 (LSM 5–7)*.

Citizens emphasised the need for government officials to lead by example and for corruption to end. Instead of looting funds, participants reported the need for government to put the nation first and improve infrastructure. Lastly the government was encouraged in future to be more proactive, plan in advance, research more and hence have solutions based on our context and possibly develop South African vaccines in response to pandemics.

*‘I only have four words for the government which are, practice what you preach.’ Group 3 (LSM 8–10)*.

*‘we need to start looking at producing our own vaccines locally because people really don’t trust generally people don’t trust anything that comes from the West.’ Group 5 (LSM 5–7)*.

## Discussion

Ten themes emerged from our qualitative analysis across all focus groups. The findings of the study shed light on the possible challenges around the implementation of policies, as well as possible solutions that may be used to enhance the public uptake of preventative measures during times of national crisis. Our results show that government strategies to combat this novel pandemic were largely appreciated by the participants. However, it was also evident that implementation may have been hampered by lack of government trust, communication by government of specific scientific information around COVID-19, and the pervasiveness of social media.

South Africa has a history of mistrust in government, which has strong links to its history of colonialism, Apartheid and corruption [26, 27, 28–30]. Nevertheless, at the onset of the pandemic, our study’s findings and other reports suggest that the president’s decisive and clear communication garnered trust amongst the nation [31]. Building upon it’s experience with other past pandemics such as the HIV/AIDS pandemic, South African COVID-19 response included the mobilization of public and private sector, following guidance from the scientific community, and conducting outreach to religious leaders and political opponents [32]. As a result, by aligning with trusted scientific and public health experts [33] and outlining all its plans, government increased risk perception and compliance amongst its citizens [34]. This led to government being a trusted source of information on the pandemic [35]. However, as the pandemic progressed our study and other surveys reported a decline in the trust and goodwill that the government had developed [36, 39]. Our participants evidenced how the corruption scandals, deployment of soldiers who assaulted civilians, and apparent disregard of COVID-19 regulations by government officials had caused suspicion of the once trusted government [37–40, 45, 46]. When a government is viewed as corrupt, it is likely that they will be perceived as being ill equipped to inform the public [41]. And in turn if the public feels the government is inadequate and untrustworthy, evidence suggests that the public are less likely to make sacrifices that require them to adhere to COVID-19 preventative measures [42]. Confirming this, some participants reported that though government had disseminated information well, they felt that government was ill equipped to do so, hence they were unwilling to comply with government restrictions. It is also well evidenced that the manner in which a message is received is generally tied to the credibility of the sender of the message. If receivers of messages disapprove of the sender of the message, they are less likely to trust the content of the message or share such messages with others [33, 50]

Agreeing with another study our participants did not report high levels of vaccine hesitancy [43]. However, participants expressed concerns around their lack of knowledge around vaccines and government’s failure to adequately educate the nation on vaccines. Participants further reported feeling forced into doing something they did not understand. Similar views were shared by respondents of the PERC study, where one in four of the 39% of respondents who said they would refuse to take the vaccine, stated the main reason was not having enough information to make a decision [5]. Additionally, participants reported being confused by scientific information on social media and criticised government’s failure to address this. Evidently, governments inability to counteract misinformation using social media, and using only mainstream media to disseminate information (which had lost favour) may have been costly [44]. Perhaps the failure of government to make use of social media platforms can be linked to the corruption scandal (in which the Minister of Health was implicated) around Digital Vibes [45], a company that was supposed to ramp up government communication around the pandemic, no doubt also using social media as a dissemination tool.

A strong and pervasive communication strategy by the government, underpinned by transparency, adequacy, and integrity would possibly have been able to positively influence the perception of the pandemic and the vaccine in the South African population [33]. Further, it may have addressed the anti-vaccine sentiment which was rife on social media and present among some nurses’ groups, who are among the most visible and accessible health care workers in South Africa. For instance, an organisation such as the Indaba nurses’ union advised its 17,000 members to boycott the vaccine because they did not trust its safety [46]. Hence, as referenced by our participants, vaccine hesitancy among nurses propagated vaccine hesitancy in their communities.

Economic and psychological impacts were experienced differently across income groups. Before the pandemic, South African low-income earners had pre-existing difficulties, namely hunger and violence, an overburdened healthcare system, a high incidence of chronic and infectious disease, and worrying rates of poverty (55.5%) and unemployment (29%) [47, 48]. Hence, there was already anxiety and depression existing within low-income settings which may have been aggravated by increased unemployment caused by the pandemic [49]. While, once comfortable middle-income participants who previously had job security, reported job losses accompanied by uncertainty and anxiety as a result of the pandemic [50]. High-income participants seemed not to be economically affected by the pandemic but reported loneliness and increased levels of anxiety; possibly associated with the lack of control the pandemic presented – something they may have previously been protected from due to their economic security. Confirming this, a participant who was a psychologist reported an increase in demand for his services. [55]

Participants also drew attention to possible solutions to the problems they highlighted. The need for community engagement and empowerment, similar to the ones conducted around HIV/AIDS were mentioned [51, 52]. In agreement with another study, the participants reported how they wanted to be represented in the decision-making process and the need for campaigns that not only inform but consult, include and empower different communities was emphasized [22]. Messaging around the pandemic, that targeted all age groups but in particular the youth (who seemed to have been left behind) was reported to be key. Following the example of countries like Singapore, the use of digital and social media, such as WhatsApp, twitter, Facebook, TikTok and other designated websites in dissemination of pandemic information was suggested [53]. Moreover, government was encouraged to proactively counter-fake news and scam alerts present on these online platforms. Social media messaging, in conjunction with the traditional SMS system, can also be used to provide information on links to websites for credible information about the latest medical information, government advisories, support programmes; as well as messages to inspire community spirit and social responsibility [53]. As misinformation was rife, participants believed that there was a need for the health literacy of citizens to be improved. Aligning with other studies, some participants believed that it was citizens responsibility to guide, educate and correct each other in a non-judgmental manner around misinformation and vaccine concerns [52, 54, 55].

Moreover, participants drew attention to the need to enhance as well as promote knowledge and understanding around the pandemic. We therefore, suggest the use of song and music videos which in the past have been reported to increase knowledge, create favourable attitudes, and change behaviours [56, 57]. This for example was done in the nineties in Zimbabwe, where Oliver Mtukudzi’s hit song ‘Neria ’ was instrumental in raising awareness around HIV/AIDS [58], and recently in Uganda Bobi Wine’s song ‘Corona virus alert’ was used to create awareness around the COVID-19 pandemic [59].

As government mistrust exists, the need to see government officials and other influential figures vaccinate and follow other measures was mentioned by participants in this study. Hence, public endorsements of pandemic by some politicians and other influencers may be beneficial. In addition, broadcasted sports events and popular TV shows can be used to raise awareness. Leveraging on the trust in the healthcare sector, the use of nurses and community health workers to educate communities may be beneficial [60]. However, to be effective ambassadors the healthcare workers need to be adequately educated and given opportunities to discuss their concerns and questions. Lastly, participants believed more could be done to improve access to vaccines in rural areas and amongst the working class. They suggested using non-traditional venues like schools or work places, which have proven successful elsewhere [61].

This study builds upon existing quantitative studies that have highlighted but have not explored in depth issues around vaccine hesitancy and pandemic perceptions. The novelty of our study lies in the fact that our results uniquely show the impacts of the pandemic in different socioeconomic groups, while highlighting possible acceptable ways to solve challenges faced. Additionally, another strength lies in having a study sample that attempts to reflect the diversity of the South African adult population. The limitations are that the online nature of the focus groups (a safer option, given the pandemic conditions) did not fully allow for body language to be captured as it was more focused on facial expressions. Lastly because the focus groups were online, we were unable to include participants from the lowest socioeconomic status (LSM 1–4), additionally those without access to equipment that includes a webcam were also unable to participate.

## Conclusion

In conclusion, with existing mistrust of government, there is need for government to leverage existing trusted sources in communities to be not only act as conduits but ambassadors of policies that government aims to implement. Furthermore, there is need to develop communication strategies that involve the scientific community, are underpinned by transparency and integrity, and aim to improve citizens’ understanding of the pandemic-relevant scientific information. Lastly, government must address the different challenges faced at different socioeconomic levels. For example, middle-income earners may need more financial support as they appeared to have lost jobs and businesses during the pandemic. While high-income earners may not need financial support, it was evident from this study that they instead need psychological support. While low-income earners may need further support to cope with additional economic stress brought about by the pandemic. Addressing these challenges in more tailored ways may also increase trust in government, which will be essential for South Africa to better navigate future pandemics and crises.

## Electronic supplementary material

Below is the link to the electronic supplementary material.

Supplementary Material 1

## Data Availability

The data used during the current study are available from the corresponding author on reasonable request.
