# Supplementary material for: Perceptions of the COVID-19 pandemic: a qualitative study with South African adults
Source: BMC Public Health. 2023 Apr 12;23:684. doi: 10.1186/s12889-023-15450-z (PMC10091320; doi:10.1186/s12889-023-15450-z)
Supplement: Supplementary file 1 — Supplementary Material 1 [file 12889_2023_15450_MOESM1_ESM.docx]

**COVID-19 Behaviour Change Think Tank**

**Focus group guide questions**

**Perceptions about COVID-19 and vaccination:**

- What do you think of the way life has changed due to the pandemic?
  - *Example: social distancing, hand sanitising/washing, wearing masks, working from home, children at home, limits on gatherings, loss of employment*
- How do you think the South African government has handled the COVID-19 pandemic?
  - *Lockdown restrictions (e.g. limits on gatherings, closing/opening of restaurants, alcohol bans), mask rules, providing the healthcare services needed*
  - *Distinguish between at the start of the pandemic, and how things are currently being handled to understand what they think about how it has been handled at different times*
- How do you think the South African government has handled the vaccination roll-out?
  - What do you think has been done well?
  - What do you think could have been / should be done better?
- What do you think about the scientific information that you have heard about COVID-19?
  - What do you think about the scientific information that you have heard about the vaccines?
- What do you think about what you have seen on social media about COVID-19?
  - What do you think about what you have seen on social media about the vaccines and vaccine roll-out?
  - *Ask about all platforms: Facebook, Instagram, Twitter, WhatsApp (friends/family/colleagues), YouTube; elicit where they are getting their information from*
- What do you think about what you have seen on other media about COVID-19?
  - What do you think about what you have seen on other media about the vaccines and vaccine roll-out?
  - *Ask about various sources: TV, radio, other online media, e.g. news websites; elicit where they are getting their information from*

**Communication:**

- Based on what you have seen and heard, what do you think about the communication about the vaccines and vaccine roll-out in South Africa?
  - What do you think has been done well?
  - What do you think could have been / should be done better?
- Is the information you have seen or heard about COVID-19 and the vaccines only from South Africa, or from other countries as well?
  - [If from other countries] How do you think it compares to information from South Africa?
- How do you decide whether information you are seeing or hearing is reliable, and not ‘fake news’?
  - *Possibly probe about “lab leak hypothesis” or their opinions about Ivermectin to explore where they get their information, who they trust etc.*
- What do you think are the main concerns about the vaccine?
  - If any you were concerned about taking the vaccine, but you got it, what encouraged you to get the vaccine?
  - If you are still not sure about taking the vaccine, what would help you change your mind?
  - If you don’t want to get the vaccine (ever), what are your main concerns about the vaccine? *[If this doesn’t get mentioned after the earlier question.]*

**Dealing with pandemics in the future:**

- What do you should be done to help us prepare for and get through future pandemics?
  - *Probe issues mentioned in the discussion*
  - Government response, behaviour changes (e.g. social distancing, masks), media, communication, vaccines

**Closing:**

- Is there anything important that you think we should have discussed that you would still like to talk about?
- Does anyone have any last thoughts to share?

*Thank you for your time and participation*
